# Metabolic Effects of *FecB* Gene on Follicular Fluid and Ovarian Vein Serum in Sheep (*Ovis aries*)

**DOI:** 10.3390/ijms19020539

**Published:** 2018-02-11

**Authors:** Xiaofei Guo, Xiangyu Wang, Ran Di, Qiuyue Liu, Wenping Hu, Xiaoyun He, Jiarui Yu, Xiaosheng Zhang, Jinlong Zhang, Katarzyna Broniowska, Wei Chen, Changxin Wu, Mingxing Chu

**Affiliations:** 1 Key Laboratory of Animal Genetics and Breeding and Reproduction of Ministry of Agriculture, Institute of Animal Science, Chinese Academy of Agricultural Sciences, Beijing 100193, China; guoxfnongda@163.com (X.G.); wangxiangyu@caas.cn (X.W.); diran@caas.cn (R.D.) liuqiuyue@caas.cn (Q.L.); huwenping@caas.cn (W.H.); hedayun@sina.cn (X.H.); 15161889913@163.com (J.Y.); 2 College of Animal Science and Technology, China Agricultural University, Beijing 100193, China; 3 Tianjin Institute of Animal Sciences, Tianjin 300381, China; zhangxs0221@126.com (X.Z.); jlzhang1010@163.com (J.Z.); 4 Metabolon, Inc., Morrisville, NC 27560, USA; KBroniowska@metabolon.com; 5 Shanghai Applied Protein Technology Co., Ltd., Shanghai 200233, China; wchen@sibs.ac.cn

**Keywords:** *FecB*, follicular fluid, ovarian vein serum, metabolic profile, ovulation rate, sheep

## Abstract

The *FecB* gene has been discovered as an important gene in sheep for its high relationship with the ovulation rate, but its regulatory mechanism remains unknown. In the present study, liquid chromatography-mass spectrometry (LC-MS) and gas chromatography-mass spectrometry (GC-MS) techniques were adopted to detect the metabolic effects of *FecB* gene in follicular fluid (FF) and ovarian vein serum (OVS) in Small Tail Han (STH) sheep. ANOVA and random forest statistical methods were employed for the identification of important metabolic pathways and biomarkers. Changes in amino acid metabolism, redox environment, and energy metabolism were observed in FF from the three *FecB* genotype STH ewes. Principal component analysis (PCA) and hierarchical clustering analysis (HCA) showed that metabolic effects of *FecB* gene are more pronounced in FF than in OVS. Therefore, the difference of the metabolic profile in FF is also affected by the *FecB* genotypes. In Spearman correlation analysis, key metabolites (e.g., glucose 6-phosphate, glucose 1-phosphate, aspartate, asparagine, glutathione oxidized (GSSG), cysteine-glutathione disulfide, γ-glutamylglutamine, and 2-hydrosybutyrate) in ovine FF samples showed a significant correlation with the ovulation rate. Our findings will help to explain the metabolic mechanism of high prolificacy ewes and benefit fertility identification.

## 1. Introduction

A single major gene responsible for high fecundity of Booroola Merino was named the *FecB* by the Committee on Genetic Nomenclature of Sheep and Goats (COGNOSAG, 1989) [1]. Based on research studies from three groups, the *FecB* gene was finally located in the *BMPR1B* gene, which presented a mutation of A746G in its coding region and caused an amino acid substitution of glutamine into arginine in the protein sequence [2,3,4]. In Booroola ewes, the effect of the *FecB* gene increased the ovulation rate and was partially dominant for litter size [5,6]. Monget et al. reported that the diameter of the ovulatory follicle was also significantly correlated with *FecB* genotypes [7]. 

Since follicle development and ovulation in ovaries were found to be controlled by follicle-stimulating hormone (FSH) and luteinizing hormone (LH) [8], it was predicted that the *FecB* gene may impact pituitary glands by increasing the release of hormone or impact ovaries in follicular cells by increasing sensitivity to the hormone [9]. After many years of arguments, the majority of researchers agree that the *FecB* gene influences the ovaries by modulating the difference in the ovulation rate [10,11,12,13]. With regard to ovarian tissue, reproduction-related gene expression, transcriptomics, and proteomics were continuously studied in *FecB* carriers’ ewes [14,15,16]. However, little attention was paid to the effect of *FecB* on ovine follicular fluid (FF), as well as the unique microenvironment which can provide energy, nutrition, and regulatory factor for oocyte development and ovulation. The message from compounds in FF may allow an oocyte to determine its own developing fate [17]. It is well known that *FecB* carriers permitted smaller follicles to develop into the ovulatory follicle [14]. In this study, we put forward a hypothesis that follicular fluid (FF) and its related ovarian vein serum (OVS) may present differently in their composition between *FecB* gene carriers and non-carriers. 

For analyzing the quality and fate of an oocyte, metabolomics has proven to be a powerful approach for discovering the small-molecule biomarkers in FF and culture medium and has been studied in humans, cattle, and pigs [18,19,20]. Wallace et al. and Bertoldo et al. found separately through ^1^H-NMR (Nuclear Magnetic Resonance) spectroscopy that the quality and developmental potential of oocytes for humans and pigs are related to metabolic profiles in FF [20,21]. Contrasted with ^1^H-NMR spectroscopy, mass spectrometry (MS)-based metabolomics techniques have become efficient platforms due to their high sensitivity and selectivity [22,23]. In the present study, liquid chromatography–mass spectrometry (LC-MS) and gas chromatography–mass spectrometry (GC-MS) techniques were adopted to discover the metabolic effects of the *FecB* gene on FF and OVS in Small Tail Han (STH) sheep. Our findings may help explain the metabolic mechanism of high prolificacy ewes and uncover some useful biomarkers for fertility identification.

## 2. Results

### 2.1. Measurement of Ovulation Rate, Number, and Diameter for Pre-Ovulation

Without any other extra exogenous hormones that may induce superovulation in ewes, ovulation rates of three *FecB* genotype ewes were successfully detected in 51 individuals using the laparoscopy procedure after treatment of estrus synchronization with CIDR (controlled internal drug releasing) (Table 1). The mean ovulation rates (total of the two ovaries) of the ++ (wild-type for *FecB* gene), +B (heterozygote mutant for *FecB* gene), and BB (homozygous mutant for *FecB* gene) groups were 1.06 ± 0.06, 2.29 ± 0.17, and 3.06 ± 0.26, respectively. Meanwhile, the comparison of ovulation rates between any two genotype groups all showed highly significant differences (*p* ≤ 0.01). 

Before collecting FF samples, the diameter and number of pre-ovulation follicles were successfully measured in 56 individuals (Table 1). The number of pre-ovulation follicle in BB ewes with 3.00 ± 0.38 was significantly higher than 2.32 ± 0.38 in +B ewes (*p* ≤ 0.01). At the same time, the pre-ovulation follicle numbers of BB and +B ewes were all significantly higher than 1.29 ± 0.10 in ++ewes (*p* ≤ 0.01). However, the opposite situation emerged in the diameter of pre-ovulation follicles. The mean diameter in ++ewes with 7.56 ± 0.52 mm was significantly larger than 5.32 ± 0.25 mm in +B ewes (*p* ≤ 0.01), and the mean diameters of ++ and +B ewes were all significantly higher than the 4.20 ± 0.15 mm mean diameter in BB ewes (*p* ≤ 0.01). In summary, the ovulation rate, pre-ovulation follicle number, and diameter in STH ewes were greatly influenced by the *FecB* gene effect. 

### 2.2. Overview of Metabolite in Pre-Ovulation FF and OVS Samples

**Figure 1:**
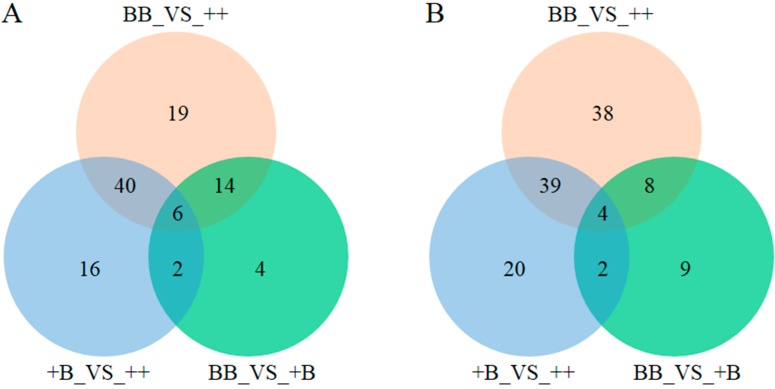
Venn diagrams for different levels of biochemical. (**A**) Venn diagrams for different levels of biochemicals in FF samples (*p* ≤ 0.05); (**B**) Venn diagrams for different levels of biochemicals in OVS samples (*p* ≤ 0.05).

Compared with the authenticated standards library of Metabolon Inc., a total of 236 and 310 known biochemical compounds were identified in ovine FF and OVS samples, respectively. All the biochemical compounds could be classified into eight super metabolic pathways (amino acid, peptide, carbohydrate, energy, lipid, nucleotide, xenobiotics, cofactors, and vitamins). With the log transformation, ANOVA was separately adopted in FF and OVS experimental group comparisons. The significantly different levels of biochemicals (*p* ≤ 0.05) in each comparison were picked out for FF and OVS samples (Figure 1). Since the *FecB* carriers have more similar biochemicals, however, BB and +B groups were accompanied by different biochemicals when compared with the wild-type groups (++) for both samples of FF and OVS. Most of the different biochemicals in FF and OVS from BB_VS_++ and +B_VS_++ were common defferecese (40 and 39). 

**Figure 2:**
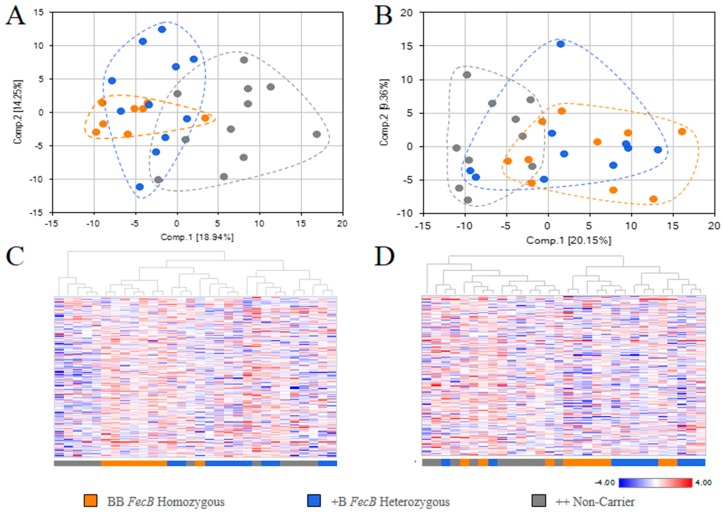
Samples clustering analysis using PCA and HCA: (**A**) PCA analysis of FF samples; (**B**) PCA analysis of OVS samples; (**C**) HCA analysis of FF samples; (**D**) HCA analysis of OVS samples.

Based on differences in overall metabolite signature in FF and OVS samples, PCA and HCA analyses were adopted to determine whether the samples can be segregated as BB, +B, and ++ groups, or whether samples from the same group can cluster together. PCA of FF revealed a separation between the group of ++ genotypes and *FecB* carrier genotypes (Figure 2A). BB samples formed the least variable population and partially overlapped with +B. HCA of FF showed that BB samples clustered together, while +B samples formed four sub-clusters interspersed by non-carriers (++) (Figure 2C). PCA and HCA analyses were also performed for OVS samples (Figure 2B,D) and showed minimal group separation in PCA and more sub-clustering in HCA based on genotypes of the *FecB* gene. Collectively, these two assessments revealed that metabolic effects associated with the *FecB* gene are more pronounced in FF than in OVS. 

### 2.3. Identification of Biomarkers in FF and OVS for FecB Gene Effect

**Figure 3:**
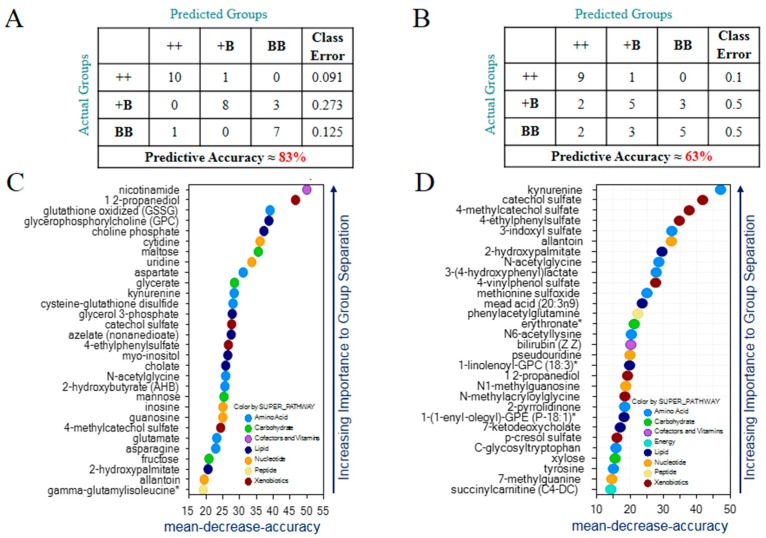
Identification of biomarkers for the *FecB* effect in FF and OVS samples. (**A**) Random forest classification in FF of BB compared to +B and ++ gave a predictive accuracy of 83%. (**B**) Random forest classification in OVS of BB compared to +B and ++ gave a predictive accuracy of 63%. (**C**) Important biochemicals identified in FF samples. (**D**) Important biochemicals identified in OVS samples.

Random forest analysis was performed for FF samples and yielded a good predictive accuracy of 83% (Figure 3A); random chance usually yields 33%. The top 30 ranking biochemicals that contributed to the differentiation are presented in Figure 3C. The process involved redox homeostasis (e.g., glutathione oxidized (GSSG), cysteine-glutathione disulfide, and 2-hydroxybutyrate (AHB)), carbohydrate metabolism (e.g., maltose, glycerate, and mannose), and lipid metabolism (e.g., glycerophosphocholine, choline phosphate, and glycerol 3-phosphate). The same analysis performed for OVS samples resulted in moderate predictive accuracy of 63% (Figure 3B) and highlighted microbiome-associated biochemicals (e.g., 4-ethylphenylsulfate, 3-(4-hydroxyphenyl)lactate, and 3-indoxyl sulfate), compounds related to nucleotide metabolism (e.g., allantoin, N1-methylguanosine, and pseudouridine) and compounds related to lipid metabolism (e.g., 2-hydroxypalmitate, mead acid(20:3n9), and 1-linolenoyl-GPc (18:3)) as strong contributors to group separation (Figure 3D). Regardless of what the samples are, the class error of the ++ group in FF and OVS samples are the lowest group. The random forest model showed weak separation power between BB and +B while also having an excellent ability to discriminate the wide-type (++) individuals from the *FecB* carriers. 

### 2.4. Changes in Energy Metabolism in FF

**Figure 4:**
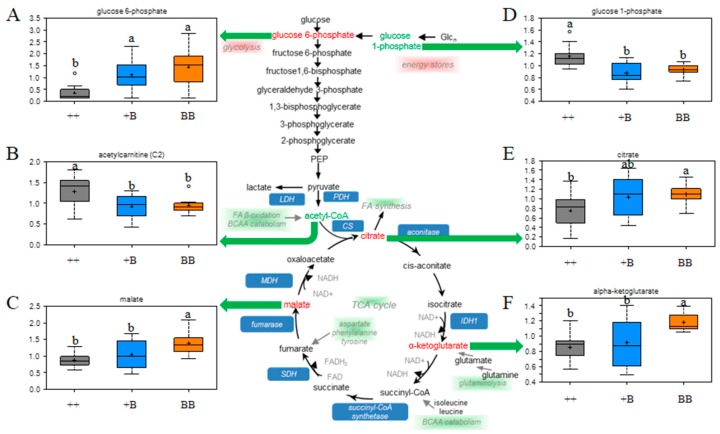
Changes in energy metabolism in FF samples. Note: a, b represent means with different superscripts in box plots have a significant difference (*p* ≤ 0.05), box plots with the ab superscript indicate no significant difference with a and b; the red font indicates that the concentration of biochemicals was elevated in BB with a significant difference (*p* ≤ 0.05), and the green font indicates that the concentration of biochemicals declined in BB with a significant difference (*p* ≤ 0.05). (**A**) Box plots for glucose 6-phosphate in FF samples; (**B**) Box plots for acetyl-CoA in FF samples; (**C**) Box plots for malate in FF samples; (**D**) Box plots for glucose 1-phosphate in FF samples; (**E**) Box plots for citrate in FF samples; (**F**) Box plots for alpha-ketoglutarate in FF samples.

FF seen as the unique microenvironment of oocyte development could provide substrates to meet energy requirements. In this study, glucose 6-phosphate acted as a glycolytic intermediate, the TCA cycle intermediates (citrate, α-ketoglutarate, and malate) along with the glycolytic intermediate were elevated in FF obtained from BB as compared to ++ individuals (Figure 4). Citrate, in addition to being an intermediate of the TCA cycle, is also a substrate for fatty acid biosynthesis, while α-ketoglutarate can be used to synthesize glutamic acid. In addition, glucose 1-phosphate levels were lower, and maltose was elevated in FF from BB animals (BB vs. (++) comparison), which might indicate increased glycogenolysis or decreased storage of glycogen. Taken together, the changes observed here indicate that the use of the TCA cycle as well as possible biosynthetic pathways utilizing the cycle intermediates are different between BB and (++) animals. 

### 2.5. Higher Levels of Amino Acids in FF from BB Sheep

The levels of several amino acids were higher in FF for ewes carrying the *FecB* gene (e.g., threonine, aspartate, asparagine, lysine, cysteine, and arginine) (Table 2). These phenomena might reflect changes in amino acid homeostasis and protein synthesis/degradation rates. As a result, higher levels of amino acids may provide a more nourishing microenvironment for oocyte development. Additionally, aspartate levels were significantly elevated in FF with increased *FecB* copies (*p* ≤ 0.05). 

### 2.6. Elevation of Antioxidant Defense Capacity in FF for BB Sheep

**Figure 5:**
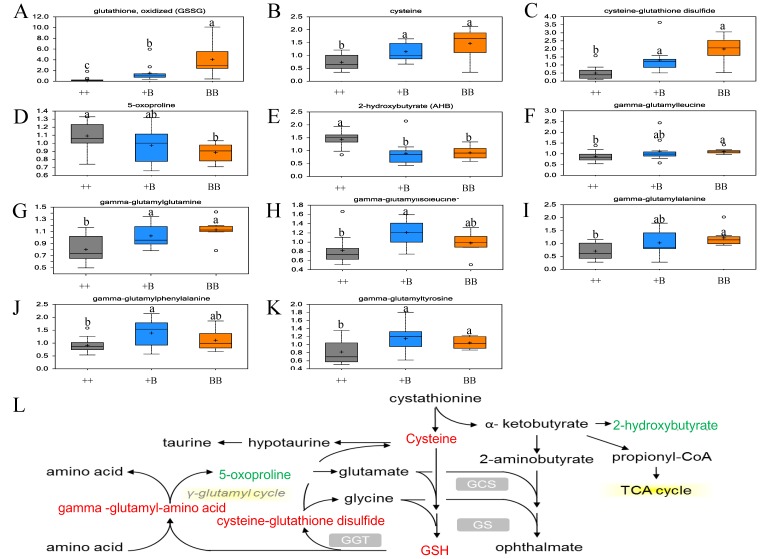
Elevation of antioxidant defense capacity in FF. Note: a, b, c represent means with different superscripts in box plots have a significant difference (*p* ≤ 0.05); box plots with ab superscript indicate no significant difference with a and b; red font indicates that the concentration of biochemicals was elevated in BB with a significant difference (*p* ≤ 0.05), and the green font indicates that the concentration of biochemicals declined in BB with a significant difference (*p* ≤ 0.05). (**A**) Box plots for glutathione, oxidized (GSSG) in FF samples; (**B**) Box plots for cysteine in FF samples; (**C**) Box plots for cysteine-glutathione disulfide in FF samples; (**D**) Box plots for 5-oxoproline in FF samples; (**E**) Box plots for 2-hydroxybutyrate in FF samples; (**F**) Box plots for γ-glutamylleucine in FF samples; (**G**) Box plots for gamma-glutamylglutamine in FF samples; (**H**) Box plots for gamma-glutamylisoleucine in FF samples; (**I**) Box plots for gamma-glutamylalanine in FF samples; (**J**) Box plots for γ-glutamylphenylalanine in FF samples; (**K**) Box plots for gamma-glutamyltyrosine in FF samples; (**L**) The biological synthesis of GSH (glutathione).

Notably, the level of oxidized glutathione (GSSG) for BB genotype in FF samples was 12.37-fold compared with the ++ genotype (*p* ≤ 0.01). Accompanied by a decrease in *FecB* copies, the level of GSSG declined (*p* ≤ 0.05) (Table 3). The levels of antioxidant defense compounds of cysteine, cysteine-glutathione disulfide, and γ-glutamyl-amino acid (including γ-glutamylalanine, γ-glutamylglutamine, γ-glutamylisoleucine, γ-glutamylleucine, γ-glutamylphenylalanine, and γ-glutamyltyrosine) were also higher in FF for BB and +B ewes vs. ++ (Table 3 and Figure 5). However, in both FF and OVS samples, 2-hydroxybutyrate (AHB) was all lower in ewes that carried the *FecB* gene. We speculate that increased antioxidant defense compounds in FF for BB ewes may have beneficial effects on more oocyte development. 

### 2.7. Subtle Alterations in Steroid Hormones for OVS

Levels of pregn steroid monosulfate in OVS showed a trend toward an increase in BB ewes as compared to ++ (0.05 < *p* ≤ 0.10). Levels of cortisone also showed a trend toward a decrease in OVS from BB animals (0.05 < *p* ≤ 0.10). Cortisone is generated by the action of 11-β-steroid dehydrogenase on peripheral cortisol. Cortisone may thus serve as a proxy for cortisol release. In this case, lower levels of cortisone may contribute to differences in stress-mediated release between *FecB* gene carriers (BB and +B) and non-carriers.

### 2.8. The Relation Between Metabolic Changes and Ovulation Rates

Spearman correlation was performed to assess the relationship between metabolic changes and ovulation rates. The normalized raw area counts data, the Scaled Imp Data, and the statistical values of ANOVA and Spearman correlation for FF and OVS samples are presented in Tables S1 and S2, respectively. Scatter plots in Figure S1 show the dependence between ovulation rate (1–4) and the level of some key metabolites (e.g., glucose 6-phosphate, glucose 1-phosphate, aspartate, asparagine, GSSG, cysteine-glutathione disulfide, γ-glutamylglutamine, and 2-hydrosybutyrate) in FF samples. The *p*-value Spearman correlation of these metabolites are less than 0.05, which means that a significant correlation and the correlations between ovulation rate and these key metabolite levels are about 0.5. 

## 3. Discussion

The discovery of the *FecB* gene, which is responsible for the ovulation rate and litter size traits in Booroola Merino sheep, assisted researchers in improving the fertility in other sheep breeds [24,25,26]. After that, several major genes for ovulation or litter size traits were found and called fecundity (*Fec*) genes, some of which include *FecX*, *FecG*, and *FecL* [27,28,29,30]. Ovulation is a sophisticated process controlled by many minor and major genes [31], and not all of the major genes found explained or worked well with regard to the ovulation rate in other high prolificacy sheep breeds. Therefore, for a long time, a variety of new technologies were adopted by researches to find new *Fec* genes. Nevertheless, no matter how powerful a *Fec* gene is, there is no difference in ovary metabolism between high and low prolificacy sheep. Equally, metabolism differences may provide another angle of view for reproduction-related gene discovery and understanding [32]. In the present study, FF and OVS from three *FecB* genotype STH ewes were selected for metabolomics analysis. Several metabolic pathways and key biomarkers were identified by ANOVA and random forest. We have verified the hypothesis that metabolites in ewes’ FF and OVS is affected by *FecB* genotypes. 

It is well known that FF is the product of the transfer of blood plasma constituents that cross the blood follicular barrier (BFB) and the secretory activity of granulosa and thecal cells [33]. Owing to the perm selectivity, it is believed that high-molecular-weight proteins are blocked by BFB, and the low-molecular-weight compounds, which could be detected by metabolomics in FF, are supposed to be similar in the OVS [34]. However, our result showed that the metabolic effects of the *FecB* gene are more pronounced in FF than in OVS. A logical deduction may be accounted for this phenomenon: the effect of the *FecB* gene on the secretory activity of granulosa and thecal cells may be strong. Campbell et al. showed that the *FecB* gene affected the response of both granulosa and theca cells to BMP, gonadotropin, and IGF-I stimulation in cultivating ovine ovarian tissue [35]. Soyun et al. found that *FecB* is expressed in cumulus–oocyte complexes (CoCs) and the loss of *FecB* in mice is associated with defective cumulus cell expansion in vitro [36]. Based on the overview of metabolomics data in the present study, we can also infer that CoCs and theca cells, which are composed the follicles, were the prime location for *FecB* affection.

Glucose is the major energy source for cells in the ovaries, and glucose concentration increases when the diameter of follicles increases [37]. Therefore, the follicles with smaller diameters measured in BB and +B can account for the lower concentration of glucose 1-phosphate, which stands for a lower level of energy storage detected in *FecB* carriers. However, as an intermediate of the TCA cycle, especially for α-ketoglutarate, which can be synthesized as glutamic acid, it was elevated in FF for BB individuals. Together with the high level of γ-glutamyl-amino acid and GSSG in *FecB* carriers, we considered that the capacity of antioxidant defense is necessary for high ovulation rate phenomenon in ewes. The elevation of antioxidant defense capacity in FF for BB sheep attracted our attention. Previously, many researchers also supported that antioxidant defense is highly correlated with oocyte developmental competence [38,39]. It is reported that low antioxidant capacity in FF accompanied poor oocyte fertilization [17,40,41]. The levels of lipid peroxidation (a proxy of oxidative stress) and the total antioxidant capacity of FF have been positively correlated with pregnancy rates in human studies [42]. The increase in oxidative stress was associated with high rates of oxidative metabolism. Elevation in the antioxidant defense has been linked to compensatory mechanisms to counteract the toxic effects of reactive oxygen species (ROS) [43]. In the current study, the increases in γ-glutamyl-amino acid, GSSG, cysteine, and cysteine-glutathione indicate the augmented antioxidant defense capacity of FF from BB ewes, which may have beneficial effects on oocyte development. 

The levels of aspartate are worth pointing out, as they were elevated significantly in FF as *FecB* copies increased, which indicates that the correlation between the aspartate level and ovulation rate in Spearman correlation analysis is significant. In human pre-ovulatory follicles, an elevation in the level of d-aspartate has been observed and correlated with the quality of oocytes and a positive outcome of in vitro fertilization [44]. In addition, d-aspartate was shown to elicit endocrine functions in rats by increasing plasma luteinizing hormone levels [45]. In our research, it was not possible to differentiate between d- and l-aspartate using this global metabolomic analysis, and specific details need to be included in further study.

Steroidogenesis is critical for follicle growth and subsequent oocyte development. Steroid hormones are synthesized from cholesterol through a series of cytochrome P450-dependent reactions [46]. In a recent study, Foroughinia et al. showed that the expression of a set of genes involved in the synthesis of steroid hormones, including Cyp19, ESR1, and ESR2, were closely associated with the number of antral follicles in ewe ovaries [47]. However, very subtle changes were observed in the levels of steroid hormones between *FecB* gene carriers and non-carriers. The levels of 17α-hydroxyprogesterone in FF also did not show a statistical difference. This might be due, at least in part, to the fact that the compound in this study was below the threshold of detection in 25–38% of the samples from the experimental groups. 

A number of microbiome-associated metabolites (e.g., 3-(4-hydroxyphenyl) lactate, indolepropionate, 3-indoxyl sulfate) that were unexpectedly elevated in OVS, also followed the same trend in the follicular fluid from BB ewes (vs ++). We predicted that the protein of FecB (BMPR1B) was also expressed in colonic epithelial cells, and aberrant BMP signaling was associated with polyposis [48,49]. Furthermore, disruption of BMP signaling in the mesenchymal cells of the colon (due to BMPR1A deletion) resulted in a toxic microenvironment with changes in extracellular matrix deposition and secretion of cytokines and growth factors [50]. Although the majority of reports focus on BMPR1A signaling in the gut, it is possible that alterations in the BMPR1B signal transduction can affect the colonic cells and thus influence the composition and/or activity of gut microbiota.

## 4. Materials and Methods

### 4.1. Animals and Grouping

Based on the TaqMan assay using the *FecB* mutation probe, three *FecB* genotypes with a total of 59 pluriparous ewes were selected from the nucleus herd of STH sheep in the southwest region of the province of Shandong in China. These ewes were all approximately 3 years old and weighed 70 kg. The same character appearance and health condition were also noted in the experimental animals. The average litter size of first and pluriparous parity in STH ewes were 200% and more than 250%. The experimental population was divided into three groups, which included 20 wild-type individuals named the ++ group, 20 heterozygote mutant individuals named the +B group, and 19 homozygous mutant individuals named the BB group. All the experimental procedures mentioned in the present study were approved by the Science Research Department (in charge of animal welfare issue) of the Institute of Animal Sciences, Chinese Academy of Agricultural Sciences (IAS-CAAS) (Beijing, China). Ethical approval on animal survival was given by the animal ethics committee of IAS-CAAS (No. IASCAAS-AE-03, 12 December 2016).

### 4.2. Collecting Data of Ovulation Rate

During the spring season, all of the experimental ewes underwent estrus synchronization. Without any interference of exogenous hormones, which may induce superovulation in ewes, CIDR (progesterone 300 mg) was inserted into the animals’ vaginas for 12 days, and 5 mL of vitamin AD was intramuscularly injected to protect the vaginal epithelium. Forage and drinking water were provided ad libitum over the duration of the experiment. After one week from the CIDR removal (luteal phase), a laparoscopy procedure was adopted for ovulation rate determination. Ovulation rate was equal to the total number of corpus luteum on both sides of an ewe’s ovary. 

### 4.3. Sample Collection and Follicle Data Measurement

Estrus synchronization mentioned above was adopted again to collect follicular fluid (FF) and ovarian vein serum (OVS). The specific protocol was as follows: after 45 h from the CIDR removal, all visible ovarian follicles with a diameter of 3.5 mm or greater were selected for follicular diameter determination. FF and OVS collection were achieved through minimally invasive surgery. The diameter of a follicle was the average value of its length (L) and width (W), which was measured by vernier caliper and the estimated volume (V) of FF was calculated using the following formula: V = 4/3 × π × [(L/2 + W/2)/2]^3^ [51]. FF samples were aspirated from non-atretic follicles with a 22-gauge needle attached to a 2 mL sterile syringe (BD, Franklin Lakes, NJ, USA) [37,52,53]. Cell debris were removed from FF by centrifuging at 4 °C at 2000 rpm for 10 min. Full blood was aspirated from ovarian veins using a 25 gauge needle with a 1 mL sterile syringe (BD, Franklin Lakes, NJ, USA) and collected in a BD SST tube. After incubating at 25 °C for 30 min aimed at clotting, OVS samples were collected from full blood and centrifuged at 4 °C at 3500 rpm for 10 min. FF samples and OVS samples were all frozen at liquid nitrogen instantly and stored at −80 °C until further analysis. Finally, 11, 11, and 8 biologic repetitions of FF were collected, and 10, 10, and 10 biologic repetitions of OVS were collected, respectively, in the ++, +B, and BB genotype groups for metabolomics analysis. 

### 4.4. LC-MS and GC-MS Analysis

Metabolite extraction and detection were performed at Metabolon Inc. (Durham, NC, USA) as previously described [54]. FF and OVS samples (100 μL) were extracted through the automated MicroLab STAR^®^ system (Hamilton Company, Bonaduz, Switzerland), and centrifuged. The resulting supernatants were analyzed by UPLC-MS/MS in a positive and negative ion mode (UPLC: Waters, Milford, MA, USA; mass spectrometer: Thermo-Finnigan LTQ, Thermo Fisher Scientific, Waltham, MA, USA, scan range, 80–1000 *m*/*z*) and by GC-MS (Thermo-Finnigan Trace DSQ fast-scanning single-quadrupole mass spectrometer, scan range 50–750 *m*/*z*).

### 4.5. Data Extraction and Compound Identification

Extraction of raw data, peak identification, and QC were performed using Metabolon’s hardware and software according to previously published methods [55]. Compounds were identified by comparison to library entries of purified standards based on the retention time/index (RI), mass to charge ratio (*m/z)*, and chromatographic data (including MS/MS spectral data) on all molecules present in the library. 

### 4.6. Metabolite Quantification and Statistical Analysis

Peaks were quantified based on the area under the curve. The normalized data for each compound were corrected into the medians of data equaling to 1.00 and making other data point proportionate. The normalized data points were also beneficial for data visualization. 

The chi-square test was used to analyze categorical variables such as the ovulation rate and the follicle number difference. One-way ANOVA was adopted to analyze continuous variables of ovulation follicle diameter and the levels of biochemicals. To assess the relationship between metabolic changes and ovulation rates, the Spearman correlation was calculated using scaled imputed log-transformed metabolite levels and ovulation rate values (R, version 3.0.2). Principal component analysis (PCA) and hierarchical clustering analysis (HCA) were used to obtain a high-level view of metabolomic datasets. PCA is a mathematical procedure that uses an orthogonal transformation to convert a set of observations of possibly correlated variables into a set of values of linearly uncorrelated variables called principle components. HCA assesses sample similarity by grouping a “hierarchy” of clustered samples and grouping metabolically similar samples close to one another. Random forest was employed to determine key biochemicals and to differentiate classification groups. Statistical analyses mentioned above were performed with the Array Studio, version 7.2 (OmicsSoft Corporation, Research Triangle Park, NC, USA) and R version 3.0.2 (http://cran.r-project.org/).

## 5. Conclusions

The results from theses global metabolomics studies comparing ewes carrying *FecB* mutation (homozygous and heterozygous) in relation to non-carriers differed in a number of metabolic readouts with a more pronounced metabolic impact of the *FecB* gene in FF than in OVS (higher number of statistically significant changes, better group separation in PCA and HCA). In FF, changes in amino acid metabolism relating to different rates of protein biosynthesis might affect the growth of the developing oocyte. Differences observed in the biochemicals involved in redox homeostasis reflected a higher oxidative pressure of rapidly dividing cells in BB animals, which indicated a greater antioxidant capacity in FF. Additionally, some key metabolites in ovine FF samples were found to significantly correlate with ovulation rate. Collectively, similar to the ovulation rate, the differences in the metabolic profile in FF is also affected by the *FecB* genotypes.

## Figures and Tables

**Table 1:** Measurements of ovulation rate, number, and diameter for pre-ovulation.

Genotype	++	+B	BB
Ovulation rate (Statistics number)	1.06 ± 0.06 ^c^ (16)	2.29 ± 0.17 ^b^ (17)	3.06 ± 0.26 ^a^ (18)
Pre-ovulation follicle number (Statistics number)	1.29 ± 0.10 ^c^ (21)	2.32 ± 0.15 ^b^ (19)	3.00 ± 0.38 ^a^ (16)
Pre-ovulation follicle diameter (Statistics number)	7.56 ± 0.52 ^a^ (27)	5.32 ± 0.25 ^b^ (31)	4.20 ± 0.15 ^c^ (36)

Note: ^a, b, c^ represent means with different superscripts in the same line have a highly significant difference (*p* ≤ 0.01). Values are means ± SEM. ++: wild-type for *FecB* gene; +B: heterozygote mutant for *FecB* gene; BB: homozygous mutant for *FecB* gene.

**Table 2:** Levels of amino acids comparing in FF.

Biochemical Name	Fold of Change			Statistical Values (*p*-Value)		
	BB/++	+B/++	BB/+B	BB/++	+B/++	BB/+B
serine	1.39	1.30	1.07	0.0572	0.0919	0.7046
threonine	1.62	1.34	1.21	0.0333	0.0752	0.5902
alanine	1.56	1.13	1.38	0.0174	0.7135	0.0372
aspartate	2.39	1.67	1.43	0.0002	0.0117	0.067
glutamate	1.45	1.15	1.27	0.0001	0.1000	0.0065
lysine	1.53	1.92	0.79	0.0081	0.0003	0.3325
tyrosine	1.24	1.33	0.93	0.0471	0.0071	0.558
tryptophan	1.25	1.16	1.08	0.0476	0.1342	0.5157
isoleucine	1.24	1.20	1.04	0.0293	0.0787	0.5377
valine	1.23	1.18	1.04	0.0356	0.101	0.5185
methionine	1.20	1.25	0.95	0.1794	0.072	0.7364
cysteine	1.99	1.55	1.28	0.0051	0.0191	0.4534
arginine	1.28	1.22	1.05	0.009	0.0177	0.6233
proline	1.20	1.18	1.02	0.039	0.0531	0.7566

Red indicates a significant difference (*p* ≤ 0.05) between the groups shown and a metabolite ratio of ≥1.00; light red indicates a narrowly missed statistical cutoff for significance 0.05 < *p* ≤ 0.10 and a metabolite ratio of ≥1.00.

**Table 3:** Levels of biochemicals related to antioxidant defense capacity comparing in FF.

Biochemical Name	Fold of Change			Statistical Values (*p*-Value)		
	BB/++	+B/++	BB/+B	BB/++	+B/++	BB/+B
glutathione, oxidized (GSSG)	12.87	4.88	2.64	0.0000	0.0001	0.0484
cysteine	1.99	1.55	1.28	0.0051	0.0191	0.4534
cysteine-glutathione disulfide	3.98	2.61	1.53	0.0000	0.0001	0.1558
γ-glutamylalanine	1.73	1.45	1.19	0.0100	0.0944	0.2479
γ-glutamylglutamine	1.40	1.28	1.10	0.0020	0.0111	0.3621
γ-glutamylisoleucine	1.20	1.48	0.81	0.1416	0.0023	0.1261
γ-glutamylleucine	1.28	1.29	0.99	0.0508	0.0886	0.6756
γ-glutamylphenylalanine	1.22	1.53	0.80	0.2842	0.0253	0.2896
γ-glutamyltyrosine	1.28	1.41	0.91	0.0413	0.0093	0.6723
2-hydroxybutyrate (AHB)	0.64	0.62	1.03	0.0082	0.0008	0.5389
5-oxoproline	0.81	0.89	0.91	0.0286	0.1369	0.3734

Red indicates significant difference (*p* ≤ 0.05) between the groups shown and a metabolite ratio of ≥1.00; light red indicates a narrowly missed statistical cutoff for significance 0.05 < *p* ≤ 0.10 and a metabolite ratio of ≥1.00; green indicates a significant difference (*p* ≤ 0.05) between the groups shown and a metabolite ratio of ≤1.00.

## Supplementary Materials

Supplementary materials can be found at http://www.mdpi.com/1422-0067/19/2/539/s1.

## Abbreviations

FF	follicular fluid
OVS	ovarian vein serum
STH	Small Tail Han
PCA	principal component analysis
HCA	hierarchical clustering analysis
GC-MS	gas chromatography-mass spectrometry
LC-MS	liquid chromatography-mass spectrometry
BFB	blood follicular barrier
ROS	reactive oxygen species
CoCs	Cumulus-oocyte complexes
CIDR	controlled internal drug releasing
