# Supplementary figures and images for: Metabolic Effects of FecB Gene on Follicular Fluid and Ovarian Vein Serum in Sheep (Ovis aries)
Source: Int J Mol Sci. 2018 Feb 11;19(2):539. doi: 10.3390/ijms19020539 (PMC5855761; doi:10.3390/ijms19020539)

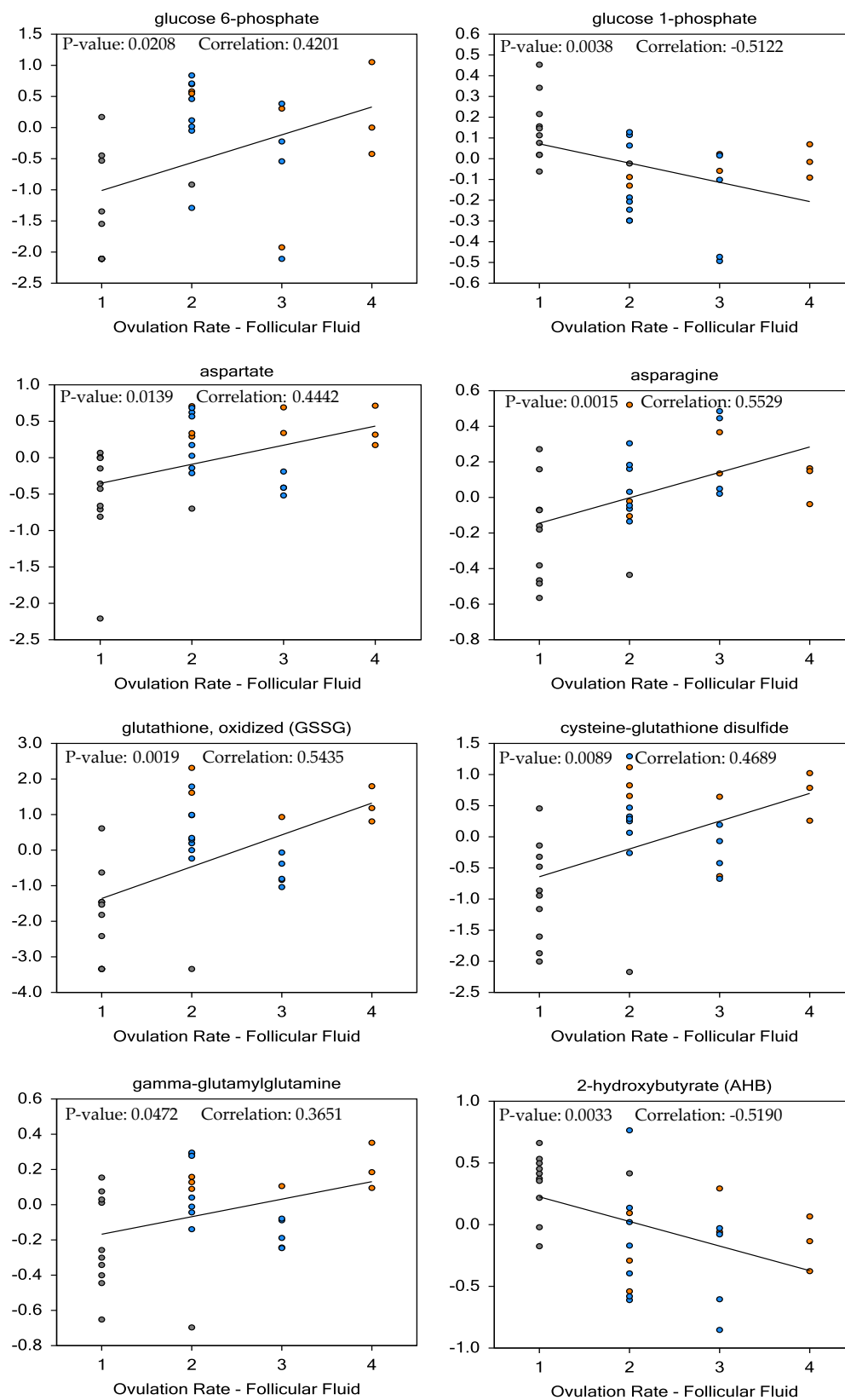

**Figure S1 Scatter plots for the correlation between ovulation rate and metabolite levels**

Supplement: Supplementary file 1 [file ijms-19-00539-s001.zip › ijms-268234-Supplementary/Figure S1 Scatter plots for the correlation between ovulation rate and metabolite levels.pdf]
